# Supplementary material for: Council of Europe Resolution on the Implementation of Pharmaceutical Care—A Step Forward in Enhancing the Appropriate Use of Medicines and Patient-Centred Care
Source: Healthcare (Basel). 2024 Jan 17;12(2):232. doi: 10.3390/healthcare12020232 (PMC10815874; doi:10.3390/healthcare12020232)
Supplement: Supplementary file 1 [file healthcare-12-00232-s001.zip › healthcare-2778282-supplementary.pdf]

COMMITTEE OF MINISTERS
COMITÉ DES MINISTRES
COUNCIL OF EUROPE
CONSEIL DE L'EUROPE

**MINISTERS' DEPUTIES** Resolutions **CM/Res(2020)3** 11 March 2020

# Resolution CM/Res(2020)3 on the implementation of pharmaceutical care for the benefit of patients and health services

*(Adopted by the Committee of Ministers on 11 March 2020 at the 1370th meeting of the Ministers' Deputies)*

The Committee of Ministers, in its composition restricted to the representatives of the States Parties to the Convention on the Elaboration of a European Pharmacopoeia (ETS No. 50),[1]

Considering that the aim of the Council of Europe is to achieve greater unity between its member States and that this aim may be pursued, *inter alia*, by the adoption of common action in the health field;

Having regard to the standard setting carried out under the Convention on the Elaboration of a European Pharmacopoeia and its Protocol (ETS No. 134), which endeavours to promote progress in every way possible, both in the social services and the related public health field, through the harmonisation of requirements for safe medicines and their safe use;

Having regard to Committee of Ministers' Resolution ResAP(93)1 on the role and training of community pharmacists, Resolution ResAP(94)1 on the rational use of medicines, and Resolution ResAP(97)2 on the development of the function of pharmacists and the adaptation of their initial training, and the need to implement them;

Recalling the measures proposed in Resolution ResAP(2001)2 concerning the pharmacist's role in the framework of health security;

Having regard to Committee of Ministers' Recommendation Rec(2006)7 on management of patient safety and prevention of adverse events in health care;

Having regard to Committee of Ministers' Recommendation CM/Rec(2008)1 on the inclusion of gender differences in health policy, which requires member States to "promote gender equality in each sector and function of the health system including actions related to health care, health promotion and disease prevention in an equitable manner", and taking into account the specific and different needs and situations of men and women as these relate to the improvement of access to and quality of health care;

Having regard to Resolution CM/Res(2016)1 on quality and safety assurance requirements for medicinal products prepared in pharmacies for the special needs of patients and Resolution CM/Res(2016)2 on good reconstitution practices in health care establishments for medicinal products for parenteral use;

Having regard to the United Nations Sustainable Development Goals and in particular, Goal 3 – Good Health and Well-Being and its targets, especially 3.D – "Strengthen the capacity of all countries, in particular developing countries, for early warning, risk reduction and management of national and global health risks";

[1] Austria, Belgium, Bosnia and Herzegovina, Bulgaria, Croatia, Cyprus, Czech Republic, Denmark, Estonia, Finland, France, Germany, Greece, Hungary, Iceland, Ireland, Italy, Latvia, Lithuania, Luxembourg, Malta, Republic of Moldova, Montenegro, Netherlands, North Macedonia, Norway, Poland, Portugal, Romania, Serbia, Slovak Republic, Slovenia, Spain, Sweden, Switzerland, Turkey, Ukraine and United Kingdom.

Having regard to the World Health Organization (WHO) Third Global Patient Safety Challenge: “Medication Without Harm”;

Having regard to *Health 2020*. *A European policy framework and strategy for the 21st century,* the policy framework for the WHO European Region and the shared goals “to significantly improve the health and well-being of populations, reduce health inequalities, strengthen public health and ensure people-centred health systems that are universal, equitable, sustainable and of high quality”;

Taking account of the proposals of WHO’s Regional Committee for Europe at its 66th Session, “Strengthening people-centred health systems in the WHO European Region: framework for action on integrated health services delivery” on organising health care providers and settings, ensuring a competent health workforce and promoting the responsible use of medicines;

Taking into account the WHO report on “The role of the pharmacist in the health care system” (1994) and the 2006 WHO handbook “Developing pharmacy practice – A focus on patient care”;

Taking into account the statement that “the mission of pharmacy practice is to contribute to health improvement and to help patients with health problems to make the best use of their medicines” by the joint International Pharmaceutical Federation (FIP)/WHO “Guidelines on good pharmacy practice: standards for quality of pharmacy services”;

Taking into account FIP policy statements and professional/scientific guidelines;

Taking into account the Pharmaceutical Group of the European Union (PGEU) publication “Pharmacy 2030: A Vision for Community Pharmacy in Europe”;

Taking into account the “European Statements of Hospital Pharmacy” of the European Association of Hospital Pharmacists (EAHP);

Considering the conclusions and recommendations of the European Directorate for the Quality of Medicines and HealthCare (EDQM) workshop entitled Quality Indicators for Pharmaceutical Care: Outcomes of the EDQM Project and Next Steps (Strasbourg, 26-27 November 2015);

Considering that medication is the most frequent intervention within a health care system and that the primary aim of all health care professionals involved in the chain of medication use should be to achieve the best possible medication outcome for the patient;

Considering that the optimisation of medication use is essential for all patient groups, for example those with chronic conditions and those receiving palliative care, and for the stewardship of antimicrobials, for sexual and reproductive care and for the overall enhancement of patient safety;

Considering that health and health care are co-produced by patients and health care professionals, the patient-centred method of pharmaceutical care is particularly important for the evolution of health services;

Considering that there is increasing evidence that the inappropriate use of medicines results in sub-optimal medication outcomes and significant health damage for patients and that it decreases the efficiency and effectiveness of health care systems; and taking into account the findings of the Council of Europe report *The gender dimension of non-medical use of prescription drugs in Europe and the Mediterranean region* (2015), which points out that women are a high-risk category for the inappropriate use of medicines;

Considering that the concept of pharmaceutical care encourages pharmacists to use their expertise and that of the pharmacy team to contribute to health promotion and illness prevention, both locally and nationally;

Considering that, in providing pharmaceutical care, pharmacists will augment the benefits of quality use of medication through relevant advice and counselling to prevent illness and to promote the health of the patient,

Recommends that the governments of the States Parties to the Convention on the Elaboration of a European Pharmacopoeia take appropriate measures to promote and implement pharmaceutical care in their respective national health systems, in line with the definition and elements described in the appendix to this resolution.

*Appendix to Resolution CM/Res(2020)3*

The appendix to Resolution CM/Res(2020)3 consists of seven sections, which define pharmaceutical care (Section 1), explain the pharmaceutical care process (Section 2), illustrate pharmaceutical care in community and hospital pharmacy practice (Sections 3 and 4), elucidate the role of pharmaceutical care in improving and maintaining the health of populations (Section 5), and formulate a number of recommendations on how to implement and promote pharmaceutical care within the health system (Sections 6 and 7).

## 1. Definition of pharmaceutical care

For the purpose of this resolution, Hepler's and Strand's definition of pharmaceutical care will be used.

According to the definition developed by Hepler and Strand, pharmaceutical care is "the responsible provision of drug therapy for the purpose of achieving definite outcomes that improve a patient's quality of life" and it "involves the process through which a pharmacist co-operates with a patient and other professionals in designing, implementing and monitoring a therapeutic plan that will produce specific therapeutic outcomes for the patient".[2]

Pharmaceutical care directly and comprehensively addresses the medication needs of patients and their quality of life. In doing so, it contributes to the overall efforts of health care professionals in providing patient-centred care and in optimising medication use. Prescribers and nurses, who, together with pharmacists, are most often involved in responding to a patient's medication needs, will benefit significantly from the introduction of pharmaceutical care. The focus of pharmaceutical care on interprofessional collaboration will also enhance team work and reduce medication-related harm, both of which are priorities for health services.

Pharmaceutical care should be envisaged as being in addition to the existing roles of pharmacists, such as the management of the supply chain and the provision of medication, including dispensing and quality assurance.

The principal elements of pharmaceutical care are the central role of the pharmacist, the patient-centred care approach, collaboration with carers,[3] prescribers and other health care professionals (integrated care), prevention, detection and resolution of medication-related problems, and taking responsibility for optimising medication use in order to improve a patient's health outcomes and quality of life.

---

[2] Hepler C.D. and Strand L.M., "Opportunities and Responsibilities in Pharmaceutical Care", *American Journal of Hospital Pharmacy* 1990; 47: 533-43.

[3] Definition: "A carer is anyone … who looks after a family member, partner or friend who needs help because of their illness, frailty, disability, a mental health problem or an addiction and cannot cope without their support" (source: National Health Service (NHS) (United Kingdom)).

## 2. Patient care and the pharmaceutical care process

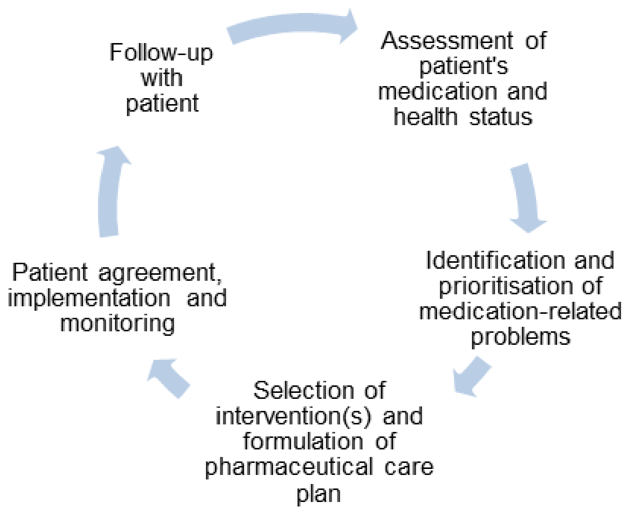


Figure 1. Pharmaceutical care process

The pharmaceutical care process consists of the following activities (Figure 1):

– Patient assessment: the main goal of the assessment is to evaluate the patient's medication-related needs and expectations, and to determine if there are any problems related to the medication therapy. This is facilitated if the pharmacist can access and contribute to the relevant records concerning the patient's health, since patients may receive medication from more than one prescriber in more than one setting. Although usually carried out face to face, some patients may not be able to take part fully in the assessment because of their circumstances and in these instances the pharmacist should work with carers and have access to the relevant records. The assessment should also aim to gather patient-related information, such as the patient's general health status, medical history including contra-indications and clinical laboratory values, management of chronic conditions and medication adherence. The assessment of the patient should be accurate and comprehensive and it should be recorded in a way that ensures it can be easily retrieved, updated and shared, if necessary.[4]

A medication review should be performed, that is, a structured evaluation of all of the patient's medicines (prescription, non-prescription, supplements and medical devices) to evaluate actual and potential medication-related problems. A medication review is necessary in particular situations and for particular patient groups when the review of a given individual's prescription would not be sufficiently comprehensive to ensure the patient's health outcomes and optimise medication use.

– Identification, resolution and prevention of medication-related problems: a medication-related problem is defined as "an event or circumstance involving drug therapy that actually or potentially interferes with desired health outcomes".[5,6] Medication-related problems are associated with, but not limited to, the medication choice, dosage and administration, adverse drug reactions, dispensing errors, prescription errors and compliance and adherence issues.[4] Medication-related problems should be assessed by the pharmacist to identify the cause(s) and, whenever possible, corrective action should be suggested. In many instances, several medication-related problems will be identified and these should be evaluated and assigned a priority according to the patient's needs and preferences.

The patient's condition and medication can increase the risk to their health, but if the patient is not experiencing any medication-related problems at the time of the evaluation, pharmaceutical care activities should focus on the prevention of problems, because changes that may have adverse health effects can still occur. Preventive action includes, but is not limited to, education and training aimed at enhancing the patient's understanding and appropriate use of their medication, regular patient follow-up (including via phone calls) and periodic interprofessional meetings during which the patient's overall care is discussed and the benefits and risks of each medication, as well as the patient's complex health care needs, are evaluated.

[4] Cipolle R.J., Morley P.C., Strand L.M., *Pharmaceutical Care Practice: The Patient-Centered Approach to Medication Management Services*, Third Edition, (2012), McGraw-Hill Education – Europe.
[5] Van Mil J.W.F., *PCNE Classification for Drug-Related Problems V9.00* (2019).
[6] Grupo de Investigación en atención farmacéutica, "Third Consensus of Granada on Drug Related Problems (DRP) and Negative Outcomes associated with Medication (NOM)", *Ars Pharmaceutica* 2007; 48(1): 5-17.

– Formulation of a pharmaceutical care plan:[4] the pharmacist should develop a tailor-made pharmaceutical care plan in co-operation with the patient, carers, prescribers and other health care professionals, as appropriate. The goal of the pharmaceutical care plan is to ensure that the medication meets the patient's needs and expectations, and that the medication contributes optimally to the effective management of the patient's condition. The pharmaceutical care plan should co-ordinate medication use from different prescribers and should enhance the patient's capability for the self-management of their condition, including the appropriate use of non-prescription medication. The pharmaceutical care plan should take account of any special needs, expectations and goals of the patient (for example, disabilities or gender-related needs) in its content and in its presentation. It should consist of several elements, highlighting each of the patient's medical conditions, their treatment and its main goals, as well as actions, responsibilities and the schedule of monitoring and follow-up. The pharmaceutical care plan should be filed in that pharmacy's records and a copy of the pharmaceutical care plan should be provided to the patient, prescribers and other health care professionals and, if appropriate, also to the family and carers. The pharmaceutical care plan is particularly important in balancing the competing and conflicting clinical priorities in polymorbid patients.

– Follow-up: regular follow-up should be performed to monitor and evaluate the patient's progress in relation to the desired outcomes included in the pharmaceutical care plan using suitable methods of communication and including periodic face-to-face consultation. The outcomes of the follow-up meetings should be documented and communicated to the patient, the prescriber and other health care professionals as necessary. As the patient's condition and health status change, this process will ensure continuity of care.

– Patient counselling, education and advice: in order for patients to optimise the use of their medication, it is necessary for them to understand the role played by their medication in the treatment of their condition. This will enhance medication adherence and safety, improve disease management and facilitate patient self-care. Counselling, education and advice should be provided in a clear and understandable manner using the most effective means (such as printed or digital patient education materials and online tools) and should be discussed and signalled/oriented so as to complement oral communication. Special consideration should be given to the psychosocial context of the patient and account should be taken of any impairments. Equally, communication with carers must take account of their role, responsibilities and capabilities and must respect the autonomy of the patient.

– Gender and health: both sex and gender have an impact on the health of women and men, on the incidence and prevalence of (specific) diseases and their identification, treatment, management and outcomes. In addition, gender in particular plays a crucial role in determining access to health care, use of the health care system and the behavioural attitudes of both patients and health care professionals. Pharmacists, as health care providers, should be encouraged to give due attention to the gender dimension in their work, by identifying where gender and/or sex-based differences exist and providing gender-sensitive care with a view to promoting equality between women and men.

Given that personal details and health information are shared in the above-mentioned steps, pharmacists should ensure that patients' privacy, dignity and the confidentiality of their data are respected throughout the whole pharmaceutical care process (for example, consultations should take place in private consultation areas, and personal health information should be handled in compliance with the relevant legal obligations and standards of practice of the profession).

### 3. Pharmaceutical care and related pharmacy services

Pharmacies provide a wide range of services to patients and the general public. Evidence of the value of pharmaceutical care continues to grow, and systematic literature reviews and meta-analysis evaluating the impact of community (general practice clinics) and hospital pharmacy services in health care have shown their positive effects on patient outcomes and economic benefits.[7]

The targeting of pharmaceutical care to specific groups of patients (diabetic patients, for example) has been systematised using guidelines and protocols to guide decision making for optimising medication use. This ensures that pharmaceutical care is standardised and of consistently high quality as it is delivered by pharmacies throughout health services.

[7] De Barra M., Scott C.L., Scott N.W., Johnston M., de Bruin M., Nkansah N., Bond C.M., Matheson C.I., Rackow P., Williams A.J., Watson M.C., "Pharmacist services for non-hospitalised patients", *Cochrane Database of Systematic Reviews* 2018; 9 (Art. No. CD013102).

The following are some examples of pharmacy services:

– Service for minor ailments and minor illnesses: prescribers and other health care professionals could refer patients with minor illnesses (for example, head lice infestations or mild dermatological conditions) to pharmacists for the necessary medication and advice instead of a general practitioner/family doctor. In some countries, community pharmacists with appropriate credentials/qualifications have also been given prescribing authority under specific conditions and based on protocols developed in collaboration with clinical teams, in order to facilitate timely access to medication and the efficient delivery of health care at an appropriate point in the health service.

– New medicine service: this service should be offered to patients who have been newly prescribed medicine for the treatment of a specific long-term condition (for example, hypertension, chronic obstructive pulmonary disease or type II diabetes). The aim is to provide them with additional support, advice and guidance in order to promote greater treatment adherence and improved disease management. The service should consist of the following steps: *a*. patient engagement (provision of advice and information when first dispensing a new medicine for long-term treatment); *b*. intervention (pharmacist-patient consultation after the first two weeks of treatment to establish the degree of adherence to the new medicine, identify any problems and explore solutions, assess the patient's needs for further information, and provide advice on a healthy lifestyle); *c*. follow-up (more structured pharmacist-patient discussions to assess progress on the actions agreed with the patient at the intervention stage, and check whether there are further questions or concerns to be addressed).[8]

– Support for complex conditions and for vulnerable people (including those with disabilities) at home or in care: the medication needs of all these groups are substantial and, if unmet, may result in negative health outcomes and the associated unnecessary health expenditure. The following services could be offered: regular multidisciplinary reviews of medication to reduce polypharmacy, optimise medication use and decrease medication-related hospital admissions; provision of advice, education and training for patients, nursing home staff, family members and carers to help improve the use and handling of medication; creation of better medication-management systems for nursing homes; domiciliary pharmacist visits to provide medication-optimisation services and, whenever applicable, to ensure a smooth transition of care between different health care settings and the patient's home.

– High-risk medication management: high-risk medications are those that have a high risk of causing significant patient harm or death if misused or used in error. Although errors may or may not be more common than with other medications, the consequences of errors with these medications can be devastating.[9] Pharmacists should contribute in a positive manner to the safe management of high-risk medications by ensuring that they are prescribed optimally, stored correctly, administered safely and used appropriately. In addition, pharmacists play a crucial role in patient safety by identifying patients on high-risk medications and ensuring that they have regular medication reviews, follow-ups of clinical targets, appropriate education and advice on medication compliance.

### 4. Services provided in the hospital setting

Hospital pharmacists contribute to the care of individual patients and they should use their expertise to develop and provide policies, guidelines and services to the hospital as a whole.

[8] NHS, "Service specification - New Medicine Service (NMS)" (2013).
[9] Australian Commission on Safety and Quality in Health Care, "High risk medicines".

As the complexity of health care increases, the process of medication use and the associated patient-care pathways become more complex in parallel. Research has shown that when patients are admitted to hospital for treatment and subsequently discharged, hospital pharmacists need to provide specific services to optimise medication use on admission, during hospitalisation and on discharge (for example, in the emergency room, in support of surgical services and at ambulatory/outpatient clinics). These services reduce medication errors, adverse drug reactions and unplanned readmissions. In most countries, hospital pharmacists are therefore central to the co-ordination of medication safety in their institutions.[10]

The following are some examples of hospital-based pharmacy services:

– Medication reconciliation: this is the process of comparing the medication prescribed to a patient with all of the medication that the patient has been taking. Medication reconciliation is done in order to avoid and/or correct medication errors (such as omissions, duplications, dosage errors, or medication interactions), to contribute to the continuity of medication treatment, and to promote effective and expeditious transmission of patient information between health care professionals. This reconciliation should be done at every transition stage of care when new medication is prescribed or existing prescriptions are rewritten (that is, on patient admission, internal transfer and discharge). The reconciliation process should comprise the following steps: *a*. the drawing up of a list of current medication (prescription and non-prescription medicines, including herbals, vitamins and food supplements); *b*. the drawing up of a list of medications to be prescribed; *c*. comparison of the two lists; *d.* a meeting with prescribers to discuss potential discrepancies, propose solutions and ensure good continuity of medical treatment; and *e*. communication of the final list to the appropriate carers and to the patient.[11]

– Individual patient care: hospital pharmacists use the pharmaceutical care process described above, which they carry out in collaboration with the other members of the health care team, according to the needs of the patient and the policies and practices of the hospital. In some countries, hospital pharmacists with appropriate credentials/qualifications who are working as part of a clinical team have been given specific prescribing authority, in a similar fashion to “advanced nurse practitioners” (experienced and highly educated registered nurses who manage the complete clinical care of their patients).

– Hospital discharge: the following activities should be performed in order to achieve a smooth transition from hospital to home: *a*. an in-depth review of discharge medication and medication reconciliation; *b*. a discharge counselling session with the patient and/or carer; *c*. provision of discharge medication (if needed), an accurate and complete list of medicines and any other additional details the patient and/or carer may need (for example, written information about medication, advice on administration and storage, instructions on how to obtain further supplies of medicines after discharge, and an explanation of the changes to therapy during hospitalisation); *d*. an update of the patient’s electronic health record and the transfer of information to all those involved in the patient’s care (for example, general practitioner, community pharmacist, other health care professionals or nursing home contact person); and *e*. an evaluation of the need for follow-up after the patient’s discharge and, if applicable, planning of follow-up action (such as home visits, phone calls).[12] Ideally, there should be a standardised, nationally approved discharge record that is sent to a general practitioner, community pharmacist and nursing home contact person (and accessible by them if available in electronic format) so that preventable readmissions may be reduced.

[10] Mueller S.K., Sponsler K.C., Kripalani S., Schnipper J.L., “Hospital-based medication reconciliation practices: a systematic review”, *Archives of Internal Medicine* 2012; 172(14): 1057-69.
[11] Sentinel Event Alert, “Using Medication Reconciliation to Prevent Errors”, *Joint Commission Journal on Quality and Patient Safety* 2006; 32(4): 230-2.
[12] The Society of Hospital Pharmacists of Australia (SHPA), “Standards of Practice for Clinical Pharmacy Services” (2016).

– Antimicrobial stewardship: this refers to a set of co-ordinated activities that include the appropriate selection, dosage, route and duration of antimicrobial therapy. It aims to optimise clinical outcomes, reduce the overuse of antibiotics, limit the selection of antimicrobial resistant strains and reduce excessive costs attributable to sub-optimal antimicrobial use.[13] Hospital pharmacists are indispensable members of multiprofessional antibiotic stewardship teams and play an important role in reviewing antibiotic treatment duration, advising on the cessation of inappropriate antibiotic treatment and educating other health care professionals on the restricted use of certain antibiotics.[14]

### 5. Services specific to public health and population health

Pharmaceutical care is a holistic approach to the patient and it recognises that medication and non-pharmacological interventions can complement or aggravate each other in their effects on the patient's health condition. Pharmacists appreciate the importance of these interventions and have used them in pharmaceutical care programmes, such as those for people with diabetes mellitus. Many non-pharmacological interventions require the patient to be the agent of change and to appreciate that health is co-produced, and therefore that working with pharmacists and other health care professionals will improve their health outcomes and quality of life.

Pharmacies are also very accessible – they are one of the few places in the health system where the public as well as patients can seek advice and services without an appointment. The accessibility and expertise of the pharmacy team make it a useful first point of contact between patients and the health and social services. This location at the heart of the community and the frequent and personal interaction enable both informal and formal approaches by the public and give them the opportunity to explore their health concerns. Pharmacies form a valuable network in which to conduct health-promotion campaigns, screening, immunisation, monitoring and signposting in support of health and wellness programmes. Health services, and population health professionals in particular, should collaborate with pharmacists to utilise the potential of pharmacies to deliver health benefits to the whole population. In many countries, voluntary health organisations already work with pharmacies to implement their campaigns.

Pharmacists should be encouraged to work with all other health care professionals and their health service to proactively provide public health services in order to improve wellness/well-being and maintain the population's health.

Pharmacists should provide screening and monitoring services. Screening services use validated tools to collect information about risk factors and sometimes offer point-of-care testing to evaluate the nature and level of risk (for example, blood glucose and lipid management services). When combined with education, advice and signposting, this results in the referral of patients in need of more comprehensive assessment and in the early detection of patients at risk. Monitoring services are provided for people receiving medication for chronic conditions, such as hypertension and hyperlipidaemia, and are useful for ensuring patient adherence to the medication and non-pharmacological interventions, as well as for detecting the progression of the condition. Both types of service should target either the general population or specific groups. Pharmacists seek to work with the health services and other health care professionals when they provide these services, and the benefits for other health care professionals and the health service would be greatly increased if the efforts of pharmacists were to receive widespread recognition, acceptance and co-operation.

Information about health and medication is easily available from a variety of sources, some authoritative and some spurious, which can lead to misunderstandings and to behaviour and lifestyle choices that are potentially harmful to health. Evidence-based, clearly expressed and consistent information about medication is needed in the health service and throughout society, as prescription medicines, non-prescription medicines and supplements are available and promoted within and across national borders. Both the public and health care professionals benefit from "medicines information services", which support them in their selection and use of medication. "Medicines information" is thus a valuable and essential tool in support of patient-care services.

[13] WHO *Global action plan on antimicrobial resistance* (2015).
[14] EAHP Position Paper on Antimicrobial Resistance (AMR), "Prudent use of antimicrobial drugs through antibiotic stewardship to ensure efficient therapy for patients with life threatening infections" (2018).

## 6. Implementation of pharmaceutical care within the health system

Health care systems need to be adapted if pharmaceutical care is to be delivered effectively. Medications are used throughout the health system and, unless their use is optimised at the system level, there is the potential for considerable medication-related harm, which will lead to the use of additional health care resources and avoidable health care expenditure.[15] Consequently, policies to promote and implement pharmaceutical care can help to address these problems. As Hepler and Strand stated: "Pharmaceutical care is a necessary element of healthcare and should be integrated with other elements."[16]

The following elements should be considered for the successful implementation of pharmaceutical care as they are interlinked, interdependent and mutually supportive:

– Policy framework: competent authorities should develop and implement a policy framework aimed at embedding pharmaceutical care in health services at regional and national levels. The aim is to ensure that pharmacists are able to contribute to the care of patients and to the design and delivery of services in every health care setting where medications are used. Reconfiguring the pharmacist's scope of practice should underpin sustainable and flexible approaches to the planning and delivery of pharmaceutical care and to the provision of services that are appropriate and responsive to population health needs. The concentration of pharmaceutical care on working with the patient and patient-related outcomes is in line with progressive developments in European health policy, such as co-production, and it represents an opportunity to maximise the utilisation of pharmacists' skills in health services.

– The role of the pharmacist within the health system: pharmacists should be recognised as providers of patient care and should be supported so that they can deliver pharmaceutical care in a professional manner.

– Access and advocacy: health care inequalities exist for many reasons, for example discrimination related to gender, sex, sexual orientation, ethnic origin or age. Pharmaceutical care should be equally available to all patients without exception and pharmacists should be given the power to advocate on behalf of disadvantaged and vulnerable patients.

– Interprofessional relationships: pharmacists, patients, prescribers and other health care professionals such as nurses are all involved in the process of medication use. In pharmaceutical care, pharmacists work in co-operation with all relevant stakeholders to ensure the efficient and safe use of medication, leading to the best possible medication outcome for the patient. In order to establish appropriate interprofessional relationships, it is necessary to establish a framework which allows the building of trust, co-operation and communication. In the pharmacist-patient relationship, both parties have a responsibility to communicate openly and to participate in the decision-making process. Patients should behave in a way that helps to achieve the desired results and, if necessary, they should be supported to do so.[17] Patients should be fully informed about the pharmacist's role and the added value of pharmaceutical care in ensuring safe and appropriate use of their medication, and they should be encouraged to make use of pharmaceutical care services.

– Structures and tools: the structures of the health care system are typically organised around primary and acute care, but inevitably this creates barriers that give rise to the need to manage medication as patients move between settings. At present, all health care professionals attempt to facilitate the transfer of information as patients move between settings, but pharmacists, like all those involved, find it difficult, time consuming and sub-optimal. Innovative tools could facilitate the provision of pharmaceutical care, such as e-prescribing, electronic health records, shared care records, clinical decision support systems and pharmacovigilance reporting software. However, these should be implemented and interoperable in all of the settings in which care is provided and medication is used. This would enhance interprofessional collaboration and, where patients' access is supported, it would empower them. All the stakeholders involved in the process of medication use are responsible for fully and routinely sharing patient information with the next provider(s) of care so that medication information moves with the patient in a timely and efficient manner.

[15] Leendertse A.J., Van Den Bemt P.M.L.A., Poolman J.B., Stoker L.J., Egberts A.C.G., Postma M.J., "Preventable hospital admissions related to medication (HARM): cost analysis of the HARM study", *Value Health* 2011; 14(1): 34-40.
[16] Hepler C.D. and Strand L.M., "Opportunities and Responsibilities in Pharmaceutical Care", *American Journal of Hospital Pharmacy* 1990; 47: 533-43.
[17] American Society of Health-System Pharmacists. "ASHP statement on pharmaceutical care" (1993).

– Resources: resources will be required to enable pharmacies to implement pharmaceutical care and to increase patients' health outcomes and safety. In most countries, in the community pharmacy sector, remuneration remains linked to the volume of prescriptions dispensed and/or the value of the products dispensed. Similarly, in the hospital sector, the pharmacy budget is frequently based on the volume of medications provided and the number of beds. However, focusing on the patient-care activities and population health services delivered by a pharmacy would be a more appropriate and effective use of pharmacists' time and expertise. It has been recognised for many years that a logical and valuable development would be the alignment of pharmacy remuneration with the provision of these activities. Pharmaceutical care, with its holistic, patient-centred and interprofessional collaborative approach, requires the investment of time and intellect, as well as increased responsibility. Given that lasting change in behaviour and practice is required to implement pharmaceutical care, the appropriate use of "nudges", incentives and deterrents in association with changes in remuneration will be necessary. Resources invested in pharmaceutical care will deliver improved patient health outcomes and increase the effectiveness and efficiency of health care delivery. Provided that pharmaceutical care is delivered in a systematic, consistent and appropriate manner, according to the needs and requirements of the health service, the introduction of a package of measures within the relevant regulatory and operational framework of care services should be evaluated.

– Quality of pharmaceutical care: the history of the EDQM at the Council of Europe is a testament to the attention that has been paid to the quality control and quality assurance of medication at every stage of its development and use. In most countries, inspection and certification of pharmacy facilities assure their quality. The development and delivery of pharmaceutical care should be subject to audit and evaluation. Ongoing research in quality assurance and the development and implementation of quality indicators should be encouraged, and those that have been piloted by the EDQM are one example of basic and generally applicable indicators that pharmacists can use to self-assess their pharmaceutical care practices.

– Health care workforce: health systems are complex, adaptive systems that need to continually adjust to the needs of patients, the variation in demand for services and the changing capability of health care technology (including medication and medical devices), and to deploy their multiprofessional workforce effectively so as to make the most of their key skills. Pharmacists should be empowered and supported through revision of their scope of practice in order to make full use of their clinical skills. In addition, pharmacies, both in the community and in hospital, are comprised of teams led by pharmacists, and the allocation of responsibilities and duties within the pharmacy should be regularly reviewed and, whenever applicable, optimised in order to enable pharmacists to maximise the time they have available to provide pharmaceutical care (for example, administrative and production tasks in the pharmacy can be delegated to pharmacy technicians in order to free up pharmacists for patient care). In all health services, it is especially important that patients are assessed by a professional with the appropriate qualifications and experience to do so. Therefore, policies and procedures should be put in place to ensure the appropriate referral of patients within the pharmacy team and to prescribers and other health care professionals when needed. These actions will facilitate the delivery of interprofessional health care and the efficient use of the skills mix within the health service.

– Education: a national framework of pharmacy qualifications, academic/undergraduate and post-academic education programmes, including continuous professional development, should be available to ensure that pharmacists acquire the knowledge, skills, motivation, confidence and capacity to deliver pharmaceutical care services in a responsible and competent manner. As pathways of care change and as new services are introduced, there is a need for assessments to ensure that pharmacists have acquired the knowledge and skills necessary to provide good pharmaceutical care. Credentials from accredited providers of education and training should recognise those who successfully complete a pharmaceutical care programme and signal to patients, prescribers and other health care professionals that the pharmacist's scope of practice has changed. Continuous professional development with appropriate accreditation and providing appropriate credentials should be used to help stimulate reflection on an individual's practice. These measures are necessary to ensure that a health service has available to it a pharmacy workforce that can contribute effectively to the promotion and implementation of national pharmaceutical care practices. Additionally, interprofessional training programmes should be developed to support health care professionals to acquire knowledge, skills and attitudes to provide patient care in a collaborative team environment.

– Evaluation: programmes and services in health systems should be subject to periodic evaluation. On the one hand, evaluation should be performed to assess the optimum delivery of pharmaceutical care and the impact of other policies and initiatives on pharmaceutical care. Assessment of the outcomes achieved and the value of pharmaceutical care are essential to the prudent use of resources. On the other hand, evaluation should also be undertaken to improve the involvement of patients and healthcare professionals in pharmaceutical care.

## 7. Promotion of pharmaceutical care

Ensuring the effective, efficient and safe use of medication is a core need of health services in all care settings. However, the adoption of new working practices requires adequate promotion and support. Therefore, pharmaceutical care should be promoted among all prescribers and stakeholders who are directly or indirectly involved in the process of medication use (such as national competent authorities, insurance funds, health care professional associations, academic institutions, prescribers, pharmacists, nurses and patients). The advancement of pharmaceutical care will depend on the extent to which patients, health care professionals and health service personnel appreciate its potential value to both themselves and the health service. Co-ownership and commitment at all levels will be required to achieve this objective.

Attention should also be paid to the fact that pharmaceutical care is of international concern, as health care and medication are provided and utilised globally and have an impact within and across national boundaries. Collaboration between national authorities, professional bodies and intergovernmental organisations should therefore be strengthened to continue to develop and share harmonised standards and best practices for the safety and quality of the process of medication use. Finally, measures should be put in place to provide all patients with equal access to high-quality pharmaceutical care services within and beyond their own country.
